# Utility of prophylactic antibiotics for preventing febrile neutropenia during cabazitaxel therapy for castration-resistant prostate cancer

**DOI:** 10.1038/s41598-021-87758-y

**Published:** 2021-04-16

**Authors:** Keitaro Watanabe, Takeo Kosaka, Hiroshi Hongo, Mototsugu Oya

**Affiliations:** Department of Urology, Keio University School of Medicine, 35 Shinanomachi, Shinjuku-ku, Tokyo, 160-8582 Japan

**Keywords:** Cancer, Medical research, Oncology, Urology

## Abstract

The aim was to investigate the efficacy of prophylactic antibiotics for the prevention of febrile neutropenia (FN) during cabazitaxel therapy for castration-resistant prostate cancer (CRPC) with G-CSF. We retrospectively studied 443 cycles of cabazitaxel therapy given to 56 patients with CRPC at Keio University Hospital between May 2012 and August 2018. Statistical analysis was conducted to determine whether the combination of prophylactic G-CSF and antibiotics was more effective in preventing FN, compared with prophylactic G-CSF alone. Prophylactic PEG-G-CSF or G-CSF was administered in all 443 cycles. Only fluoroquinolones were used as prophylactic antibiotics and were administered in 328 cycles (74.0%). FN occurred in 5 cycles (1.1%). Prophylactic antibiotics were administered in 327 cycles (74.6%) in the FN-negative group and in only 1 cycle (20.0%) in the FN-positive group. Chi-square test indicated the incidence of FN was significantly lower in the group that received prophylactic antibiotics compared with the group that did not receive prophylactic antibiotics (*P* = 0.017). Compared with prophylactic G-CSF alone, prophylactic G-CSF and antibiotics significantly suppressed the occurrence of FN.

## Introduction

Cabazitaxel for metastatic castration-resistant prostate cancer (mCRPC) has been recommended as second-line chemotherapy after docetaxel. A phase III trial (i.e., TROPIC study) demonstrated improved overall survival in patients with CRPC after or during docetaxel-containing therapy^1^. However, cabazitaxel therapy often has a high risk for hematologic toxicity, such as neutropenia. According to the TROPIC study, World Health Organization (WHO) grade 3 or higher neutropenia occurred in 82% and febrile neutropenia (FN) occurred in 8% during cabazitaxel therapy^1^. On the other hand, a phase I study in Japan reported high rates of WHO grade 3 or higher neutropenia in 100% and FN in 54.5%^2^. The American Society of Clinical Oncology (ASCO) guideline recommended prophylactic administration of granulocyte-colony-stimulating factor (G-CSF) for regimens that have a reported FN incidence of ≥ 20%^3^. In Japan, a phase IV trial (i.e., PEGAZUS study) revealed a 9.5% rate of FN among all patients (*N* = 21) who were administered prophylactic polyethylene glycol (PEG)-G-CSF^4^. These results indicated that prophylactic G-CSF was effective for prevention of FN.

To date, in Japan, the reported incidence of WHO grade 3/4 FN ranged from 16.3 to 30.6% and prophylactic G-CSF administration was reported in 65.9% to 95%^5–8^. Our previous research on the same patient population (*N* = 41) reported a 7.3% incidence rate for FN^9^; this rate was slightly lower, compared with the rates in the other reports in Japan. On hindsight, we deemed that the reason for this low rate was the administration of prophylactic antibiotics, with informed consent, for patients in whom the risk for FN was considered high. Our previous study revealed that patients aged ≥ 75 years and those with a history of radiation therapy were at high risk for FN when treated with docetaxel for CRPC^10^.

On the other hand, the ASCO guideline recommended administration of prophylactic antibiotics only for patients who were expected to have < 100 neutrophils/μL for > 7 days^11^.

In cabazitaxel therapy, the utility of prophylactic administration of antibiotics for the prevention of FN is unclear. The aim of this study was to reveal the efficacy of prophylactic antibiotics for the prevention of FN during cabazitaxel therapy with G-CSF.

## Patients and methods

We retrospectively researched a total of 443 cycles of cabazitaxel therapy given to 56 patients who had progressive castration-resistant prostate adenocarcinoma during docetaxel therapy at Keio University Hospital between May 2012 and August 2018. The work described has been carried out in accordance with the Declaration of Helsinki for experiments involving humans. Obtaining informed consent was exempted because our study design was retrospective and all identifying information was removed. For each cycle, we retrospectively investigated the cabazitaxel dose, prophylactic antibiotics, prophylactic administration of G-CSF, and the development of FN. Almost all patients were given intravenous cabazitaxel at 20–25 mg/ m^2^ (median 22.5 mg/ m^2^) with prednisone 10 mg/ day every 3–4 weeks. All patients were administered prophylactic PEG-G-CSF or G-CSF. Only fluoroquinolones were used as prophylactic antibiotics. Subcutaneous injection of pegfilgrastim (3.6 mg) was administered as PEG-G-CSF for 411 cycles. Subcutaneous injection of lenograstim (100 μg) daily was administered as G-CSF for 32 cycles. At the beginning of the enrollment period, pegfilgrastim was not approved in Japan, so we administered lenograstim daily. But after approval, we have unified lenograstim with pegfilgrastim. Lenograstim was administered to 8 patients for 32 cycles. Regarding the administration of prophylactic antibiotics, we administered ciprofloxacin 200 mg orally twice daily or levofloxacin 500 mg orally once daily or sitafloxacin 50 mg orally twice daily for 7 days. In Japan, a phase IV trial (i.e., the PEGAZUS study)^4^ determined that the median time to nadir in the Japanese population was 8 days (range 7–9 days), so it is considered necessary to cover the median time to nadir on the administration start date. We administered prophylactic G-CSF on day 2–4 and prophylactic antibiotics on day 4–5 to cover the nadir period.

According to the Infectious Diseases Society of America (IDSA), we defined neutropenia as an absolute neutrophil count of < 1000/ μL and severe neutropenia as an absolute neutrophil count of < 500/ μL. We defined FN as a single oral temperature of ≥ 38.3 °C or a sustained temperature of ≥ 38.0 °C for more than 1 hour^12^.

The continuous variables of the FN-positive and FN-negative groups were compared using the chi-square test. For multivariate analysis, logistic regression was used. For all statistical analyses, tests were one-sided and *P* < 0.05 was considered to indicate statistical significance. All statistical analyses were performed using the Statistical Package of the Social Sciences, version 25.0 (SPSS, Chicago, IL, USA).

### Ethics declaration

All procedures performed in studies involving human participants were in accordance with the ethical standards of the institutional and/or national research committee and with the Declaration of Helsinki (1964) and its later amendments or comparable ethical standards. The study was approved by the Institutional Ethics Board of Keio University Hospital (No. 20150159).

### Consent to participate and publication

This study was approved by the Institutional Ethics Board of Keio University Hospital (No. 20150159), which exempted obtaining informed consent because our study design was retrospective and all identifying information was removed.

## Results

### Patient characteristics

All patient characteristics are shown in Table 1. All patients are Japanese and received docetaxel therapy for at least one cycle. The median age was 71.0 years (range 46–85 years). The Eastern Cooperative Oncology Group performance status (ECOG PS) score at the time of introduction of cabazitaxel was 0 in 83.9% and 1/ 2 in 16.1%. The median baseline prostate-specific antigen level was 87.7 ng/ mL (range 0.17–11,660 ng/ mL). The major sites of metastases were the bone (96.4%) and lymph nodes (58.9%). Prior docetaxel was given for a median number of 7 cycles (range 1–43) cycles. Cabazitaxel was administered for a total number of 443 cycles. The administration protocol for cabazitaxel generally followed the phase III TROPIC trial^1^ and the TED 11,576 study in Japan^13^. The dose of cabazitaxel was ≤ 20 mg/m^2^ in 37.9%, 22.5 mg/m^2^ in 29.6%, and 25 mg/m^2^ in 32.4%. The reasons for dose reduction to ≤ 20 mg/m^2^ were old age, reduction of PS during the cabazitaxel administration cycle, and grade 3/4 adverse effect (AE) in the previous cycle. Prophylactic PEG-G-CSF or G-CSF was administered in all cycles, whereas prophylactic antibiotics were administered in 328 cycles (74.0%). No AE was observed after prophylactic antibiotics. FN occurred in 5 cycles (1.1%) in 4 patients. Cabazitaxel in combination with prednisone was well tolerated. Table 2 shows the background of all patients between the group of patients with all cycles of prophylactic antibiotics (group A), and the group of patients with cycles without prophylactic antibiotics (group B). In the group receiving antibiotics in all cycles, the dose of cabazitaxel was tend to be reduced and hemoglobin (Hb) tended to be low.

**Table 1:** Characteristics of patients treated with cabazitaxel.

Characteristics	Value (*N* = 56)
**Cabazitaxel**	
Median age (range)	71 (46–85)
< 75 years (%)	46 (82.1)
≥ 75 years (%)	10 (17.9)
Total cycles (median, range)	443 (5, 1–46)
Dose, mg/m^2^, (%)	22.5 (25–10)
25	144 (32.4)
22.5	131 (29.6)
≤ 20	168 (37.9)
Cycles with prophylactic antibiotics (%)	328 (74.0)
Cycles with G-CSF or PEG-G-CSF (%)	443 (100)
Cycles in which FN occurred (%)	5 (1.1)
**ECOG PS at introduction of cabazitaxel, *N* (%)**	
0	47 (83.9)
1, 2	9 (16.1)
PSA at base line, ng/mL, median	87.7 (0.17–11,660)
**Site involved,* N*** **(%)**	
Bone	54 (96.4)
Lymph node	33 (58.9)
Visceral metastases	18 (32.1)
**Prior 2nd AR targeting lines,** ***N*** **(%)**	
Enzalutamide/Abirateron	47 (83.9)/33 (58.9)
Total prior docetaxel cycle, median (range)	7 (1–43)
Prior radiation therapy (%)	16 (22.5)
Hb at base line, g/dL, median (range)	11.4 (8.0–14.8)
NLR at base line, median (range)	4.3 (1.2–16.0)
AMC at base line, /μL, median (range)	374 (105–941)
ALP at base line, IU/I, median (range)	276 (113–3146)
LDH at base line, U/I, median (range)	224 (148–997)

G-CSF, granulocyte-colony stimulating factor; PEG-G-CSF, polyethylene glycol-granulocyte-colony stimulating factor; FN, febrile neutropenia; ECOG PS, Eastern Cooperative Oncology Group performance status; PSA, prostate-specific antigen; AR, androgen receptor; Hb, hemoglobin; NLR, Neutrophil to Lymphocyte ratio; AMC, absolute monocyte count; ALP, alkaline phosphatase; LDH, lactate dehydrogenase.

**Table 2:** Characteristics of patients between the group with all cycles with prophylactic antibiotics and the group with cycles without prophylactic antibiotics.

Characteristics	Group A	Group B	p value
**Cabazitaxel**			
Patients (N = 56)	37	19	
Median age (range)	71 (49–85)	70 (46–79)	0.318
< 75 years (%)	31 (83.8)	15 (78.9)	0.72
75 years (%)	6 (16.2)	4 (21.0)	
Dose, mg/m^2^, (%)			0.010
25	15 (40.5)	15 (78.9)	
< 25	22 (59.5)	4 (21.1)	
**FN**			0.108
Yes	1 (2.7)	3 (15.8)	
No	36 (97.3)	16 (84.2)	
**ECOG PS**			1
0	31 (83.8)	16 (84.2)	
1, 2	6 (16.2)	3 (15.8)	
PSA at base line, ng/mL, median (range)	138.6 (0.35–3120)	37.69 (0.17–11,660)	0.236
**Site involved**			
Bone (%)	36 (97.3)	18 (94.7)	0.568
Lymph node (%)	25 (67.6)	8 (42.1)	0.061
Visceral metastases (%)	13 (35.1)	5 (26.3)	0.361
**Prior 2nd AR targeting lines**			
Enzalutamide (%) /Abirateron (%)	34 (91.9) / 21 (56.8)	13 (68.4) / 12 (63.1)	0.033/0.43
Total prior docetaxel cycle median (range)	7 (1–43)	6 (1–27)	0.33
Prior radiation therapy	11 (29.7)	5 (26.3)	0.524
Hb at base line, g/dL, median (range)	10.9 (8.0–14.8)	12 (9.4–14.7)	0.037
NLR at base line, median (range)	4.2 (1.8–16.0)	5 (1.2–11.0)	0.684
AMC at base line, /μL, median (range)	372 (105–940)	380 (199–941)	0.802
ALP at base line, IU/I, median (range)	306 (113–3146)	261 (121–1793)	0.511
LDH at base line, U/I, median (range)	256 (148–997)	220 (163–848)	0.229

Group A, patients with all cycles of prophylactic antibiotics. Group B, patients with cycles without prophylactic antibiotics. FN, febrile neutropenia; ECOG PS, Eastern Cooperative Oncology Group performance status; PSA, prostate-specific antigen; AR, androgen receptor; Hb, hemoglobin; NLR, Neutrophil to Lymphocyte ratio; AMC, absolute monocyte count; ALP, alkaline phosphatase; LDH, lactate dehydrogenase.

### Efficacy of FN prevention during cabazitaxel therapy

**Figure 1:**
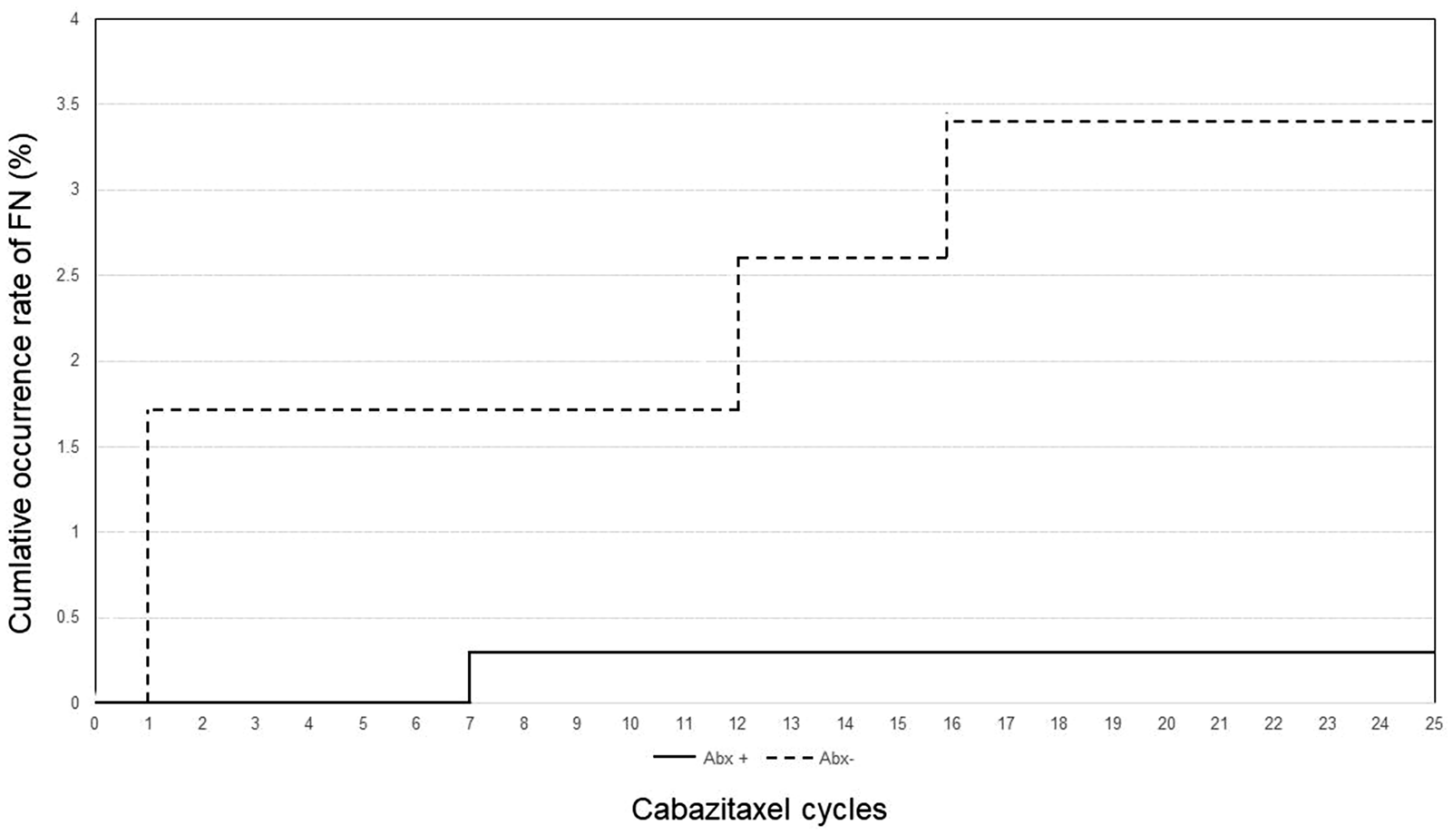
The number of cabazitaxel cycles and the cumulative occurrence rate of FN occurrence cycles between the group of cycles with prophylactic antibiotics and cycles without prophylactic antibiotics.

Table 3 shows the comparison of the administration cycles of cabazitaxel according to the development of FN. Among the 443 cycles of cabazitaxel administered, FN occurred in 5 cycles in 4 patients. In all 4 patients, prophylactic G-CSF was administered for patient. Figure 1 shows the number of cabazitaxel cycles and the cumulative number of FN occurrence cycles between the group of cycles with prophylactic antibiotics and cycles without prophylactic antibiotics.

**Table3:** Comparison of the administration cycles of cabazitaxel according to the development of FN.

	FN negative	FN positive	*P* value
Total cycles of cabazitaxel	438	5	
Median cycles each patient	5	7	0.044
**Age (years)**			0.595
< 75	366	4	
≥ 75	72	1	
**Dose (mg/m**^**2**^**)**			
25	141	3	0.415
22.5	130	1	
≤ 20	167	1	
25	141	3	0.197
< 25	297	2	
**ECOG PS at introduction of cabazitaxel**			0.670
0	404	5	
1,2	34	0	
**Prophylactic antibiotics**			
Yes	327	1	0.017
No	111	4	
WBC nadir median during FN (/μL)		1000 (400–1300)	
Neut. Nadir median during FN (/μL)		50 (42–195)	

ECOG PS, Eastern Cooperative Oncology Group performance status; WBC, white blood cell; Neut., neutrophils; FN, febrile neutropenia.

Prophylactic antibiotics were administered in 327 cycles (74.6%) in the FN-negative group and in only 1 cycle (20.0%) in the FN-positive group. The patient in whom FN occurred with prophylactic antibiotics was 71 years old. FN occurred on day 6 of the 7th cycle of cabazitaxel. PEG-G-CSF was administered on day 3, and oral ciprofloxacin administration was started on day 3 for 7 days. He had cough and sputum. Chest X-ray film revealed a consolidation in the left lower lung zone, suggesting pneumonia. *Enterobacter cloacae, Klebsiella pneumoniae, and Citrobacter freundii* complex were detected in sputum cultures. Blood cultures were negative. Ciprofloxacin-resistant bacteria were not detected. Meropenem (1 g) was administered intravenously thrice a day. He was discharged on the 10th day of illness after the recovering from pneumonia.

Dose reduction was classified as < 25 mg, but no significant difference was observed between the two groups. The patients were grouped by age (≥ 75 years or < 75 years) and by the ECOG PS at the time of introduction (0 or 1, 2). No significant difference was observed. Results of the analysis revealed that prophylactic antibiotics significantly reduced the FN rate (*P* = 0.017).

## Discussion

Our results showed a significantly lower FN incidence in the group that received prophylactic antibiotics than in those that did not (*P* = 0.017), but the dose, age, and ECOG PS were not. Prophylactic G-CSF has been shown to reduce the incidence of FN, which can be burdensome for patients, especially those who go through a serious course. Therefore, keeping the incidence of FN as low as possible is necessary. Although the ASCO guideline recommended prophylactic antibiotics only for patients expected to have < 100 neutrophils/ μL for > 7 days^3^, we considered that concomitant administration of antibiotics and G-CSF would be a superior method.

The use of prophylactic antibiotics has been most commonly reported in the hematologic oncology field. A systematic review of 109 trials (*N* = 13,579) reported that prophylactic antibiotics helped reduce overall mortality (RR = 0.66; 95% CI 0.55–0.79) and infection-related mortality (RR = 0.61; 95% CI 0.48–0.77)^14^. Most of these reports were on chemotherapy for hematologic malignancies. Only few reports have compared prophylactic G-CSF with antibiotics and prophylactic G-CSF alone for other solid carcinomas; we summarized these reports in Table 4^15–19^. Many of these studies reported a reduced incidence of FN by concomitant use of antibiotics. One report stated that there was no significant difference in the incidence of FN between the G-CSF plus prophylactic antibiotic and the G-CSF alone groups; however, G-CSF alone had very low FN incidence in 1 of 23 patients^17^. One study reported no significant difference when the neutropenia grade was 3 or less, but in the group with grade 4 neutropenia, infections were significantly suppressed (61% vs. 22%, *P* = 0.002)^19^. In Japan, the rate of WHO grade 3/4 neutropenia due to cabazitaxel was high^2^, and prophylactic antibiotics are expected to be efficacious. For solid carcinoma, one study on 161 patients who received cyclophosphamide, doxorubicin, and etoposide regimen for small cell lung carcinoma showed 55% reduction of FN incidence with the administration of G-CSF plus prophylactic antibiotics than of G-CSF alone^18^. There were very few reports with similar comparisons for each solid carcinoma or regimen.

**Table 4:** Comparison of the prophylactic G-CSF plus antibiotics and prophylactic G-CSF alone for cancer in other regions.

Author	Year	*N*	Type of cancer	Incidence of FN or infection
1: Maiche et al.^19^	1993	59	ML, Breast cancer, HL and so on	22% 61% *(P* = 0.002) (WHO grade 4)
2: Tjan-Heijnen et al.^18^	2003	244	SCLC	7% :15%
3: Lalami et al.^17^	2004	48	Breast cancer, Colorectal cancer, Head and neck cancer, Lung cancer, and so on	0% : 4.3% (*P* = 1)
4: Eleutherakis-Papaiakovou et al.^16^	2010	157	MM, other hematologic malignancy and on HDT with ASCT	56.2% : 91.2% (*P* < 0.001)
5: Fen et al.^15^	2014	38	AML	0.4 : 0.9 (*P* < 0.001)

ML, malignant lymphoma; HL, Hodgkin’s lymphoma; SCLC, small cell lung cancer; MM, multiple myeloma; HDT, high-dose chemotherapy, ASCT, autologous stem-cell transplantation; AML, acute myelogenous leukemia.

There were several limitations in our study. First, the study design was retrospective and involved a relatively small population of Japanese patients with mCRPC. Therefore, the findings obtained in this study should be verified in a prospective study that includes other ethnic groups.

Second, the adaptation of the results is limited to the Japanese population. The incidence of FN in the Japanese population was 9.5% despite the prophylactic use of G-CSF^4^. On the other hand, according to the TROPIC study, the incidence of FN was 8% without prophylactic G-CSF. It is assumed that Japanese people are at high risk of FN.

Third, since the number of FN cycles is less and the statistics are unstable, multivariate analysis could not be performed. The results of multivariate analysis are summarized in Supplemental Table 1 as reference values.

Fourth, a possibility of the emergence of resistant bacteria with the administration of prophylactic antibiotics is concerning. In our study, none of the patients developed resistant bacterial infection, and none of the reports listed in Table 4 showed apparent appearance of resistant bacteria^15–19^. However, there was one study that reported increased resistant bacterial colonization with prophylactic fluoroquinolone^20^. The emergence of resistant strains is described in the double-blind, randomized controlled trial that investigated the significance of prophylactic antibiotics in the treatment of myeloma. There was no significant difference in the carriage rates of expanded-spectrum β-lactamase (ESBL) Gram-negative coliforms, and methicillin-resistant *Staphylococcus aureus (*MRSA) between the placebo group and the levofloxacin group^21^. It would be necessary to keep in mind the possible emergence of resistant bacteria and to refrain from unnecessary long-term use of antibiotics.

## Conclusion

Compared with G-CSF alone, prophylactic administration of G-CSF preparations plus antibiotics significantly reduced the occurrence of FN during cabazitaxel therapy for CRPC.

## Supplementary Information

Supplementary Information

## Data Availability

The datasets analyzed during the current study are not publicly available because these contain information that could compromise the privacy of the study participants but are available from the corresponding author on reasonable request.

## References

[1] de Bono JS (2010). Prednisone plus cabazitaxel or mitoxantrone for metastatic castration-resistant prostate cancer progressing after docetaxel treatment: A randomised open-label trial. Lancet (London, England).

[2] Nozawa M (2015). Japanese phase I study of cabazitaxel in metastatic castration-resistant prostate cancer. Int. J. Clin. Oncol..

[3] Smith TJ (2015). Recommendations for the use of WBC growth factors: American Society of Clinical Oncology Clinical Practice Guideline Update. J. Clin. Oncol..

[4] Kosaka T (2019). Impact of pegfilgrastim as primary prophylaxis for metastatic castration-resistant prostate cancer patients undergoing cabazitaxel treatment: An open-label study in Japan. Jpn. J. Clin. Oncol..

[5] Yamamoto T (2020). Safety and efficacy of cabazitaxel in Japanese patients with castration-resistant prostate cancer. Prostate Int..

[6] Shiota M (2019). Efficacy and safety of 4-weekly cabazitaxel for castration-resistant prostate cancer: A multi-institutional study. Cancer Chemother. Pharmacol..

[7] Suzuki K (2019). Safety and efficacy of cabazitaxel in 660 patients with metastatic castration-resistant prostate cancer in real-world settings: Results of a Japanese post-marketing surveillance study. Jpn. J. Clin. Oncol..

[8] Terada N (2019). The efficacy and toxicity of cabazitaxel for treatment of docetaxel-resistant prostate cancer correlating with the initial doses in Japanese patients. BMC Cancer.

[9] Kosaka T, Shinojima T, Morita S, Oya M (2018). Prognostic significance of grade 3/4 neutropenia in Japanese prostate cancer patients treated with cabazitaxel. Cancer Sci..

[10] Shigeta K (2015). Predictive factors for severe and febrile neutropenia during docetaxel chemotherapy for castration-resistant prostate cancer. Int. J. Clin. Oncol..

[11] Taplitz RA (2018). Antimicrobial prophylaxis for adult patients with cancer-related immunosuppression: ASCO and IDSA clinical practice guideline update. J. Clin. Oncol..

[12] Taplitz RA (2018). Outpatient management of fever and neutropenia in adults treated for malignancy: American Society of Clinical Oncology and Infectious Diseases Society of America Clinical Practice Guideline Update. J. Clin. Oncol..

[13] Mukai H (2014). Phase I dose-escalation and pharmacokinetic study (TED 11576) of cabazitaxel in Japanese patients with castration-resistant prostate cancer. Cancer Chemother. Pharmacol..

[14] Gafter-Gvili A (2012). Antibiotic prophylaxis for bacterial infections in afebrile neutropenic patients following chemotherapy. Cochrane Database Syst. Rev..

[15] Feng X (2014). Prophylactic first-line antibiotics reduce infectious fever and shorten hospital stay during chemotherapy-induced agranulocytosis in childhood acute myeloid leukemia. Acta Haematol..

[16] Eleutherakis-Papaiakovou E (2010). Prophylactic antibiotics for the prevention of neutropenic fever in patients undergoing autologous stem-cell transplantation: Results of a single institution, randomized phase 2 trial. Am. J. Hematol..

[17] Lalami Y (2004). A prospective randomised evaluation of G-CSF or G-CSF plus oral antibiotics in chemotherapy-treated patients at high risk of developing febrile neutropenia. Support. Care Cancer.

[18] Tjan-Heijnen VC (2003). Economic evaluation of antibiotic prophylaxis in small-cell lung cancer patients receiving chemotherapy: An EORTC double-blind placebo-controlled phase III study (08923). Ann. Oncol..

[19] Maiche AG, Muhonen T (1993). Granulocyte colony-stimulating factor (G-CSF) with or without a quinolone in the prevention of infection in cancer patients. Eur. J. Cancer.

[20] Lew MA (1991). Prophylaxis of bacterial infections with ciprofloxacin in patients undergoing bone marrow transplantation. Transplantation.

[21] Drayson MT (2019). Levofloxacin prophylaxis in patients with newly diagnosed myeloma (TEAMM): A multicentre, double-blind, placebo-controlled, randomised, phase 3 trial. Lancet Oncol..

