# Supplementary material for: Utility of prophylactic antibiotics for preventing febrile neutropenia during cabazitaxel therapy for castration-resistant prostate cancer
Source: Sci Rep. 2021 Apr 16;11:8367. doi: 10.1038/s41598-021-87758-y (PMC8052377; doi:10.1038/s41598-021-87758-y)
Supplement: Supplementary file 1 — Supplementary Information [file 41598_2021_87758_MOESM1_ESM.docx]

Supplemental Table 1. Multivariate analysis of factors that may prevent FN

| Factors |  | Logistic regression analysis | |
| --- | --- | --- | --- |
|  | univariate | multivariate | |
|  | *P* value | *P* value | HR (95% Cl) |
| age(years) | 0.595 | 0.231 |  |
| <75 |  |  |  |
| ≥75 |  |  |  |
| dose (mg/m^2^) | 0.197 | 0.467 |  |
| 25 |  |  |  |
| <25 |  |  |  |
| ECOG PS at introduction of cabazitaxel | 0.67 | 0.617 |  |
| 0 |  |  |  |
| 1, 2 |  |  |  |
| prophylactic antibiotics | 0.017 | 0.028 | 0.085 (0.009–0.767) |
| yes |  |  |  |
| no |  |  |  |
|  | | | |
| ECOG PS, Eastern Cooperative Oncology Group performance status | | | |
